# Sustained Exendin-4 Secretion through Gene Therapy Targeting Salivary Glands in Two Different Rodent Models of Obesity/Type 2 Diabetes

**DOI:** 10.1371/journal.pone.0040074

**Published:** 2012-07-13

**Authors:** Giovanni Di Pasquale, Ilaria Dicembrini, Laura Raimondi, Claudio Pagano, Josephine M. Egan, Andrea Cozzi, Lorenzo Cinci, Andrea Loreto, Maria E. Manni, Silvia Berretti, Annamaria Morelli, Changyu Zheng, Drew G. Michael, Mario Maggi, Roberto Vettor, John A. Chiorini, Edoardo Mannucci, Carlo M. Rotella

**Affiliations:** 1 Molecular Physiology and Therapeutics Branch, National Institute of Dental and Craniofacial Research, National Institutes of Health, Bethesda, Maryland, United States of America; 2 Section of Endocrinology, Department of Clinical Pathophysiology, University of Florence, Florence, Italy; 3 Department of Pharmacology, University of Florence, Florence, Italy; 4 Endocrine-metabolic Laboratory, Department of Medical and Surgical Sciences, University of Padua, Padua, Italy; 5 Diabetes Section, National Institute on Aging and Health, Baltimore, Maryland, United States of America; 6 Section of Histology, Department of Anatomy, University of Florence, Florence, Italy; 7 Sexual Medicine and Andrology Unit, Department of Clinical Physiopathology, University of Florence, Florence, Italy; 8 Diabetes Agency, Careggi Teaching Hospital, Florence, Italy; University of Santiago de Compostela School of Medicine - CIMUS, Spain

## Abstract

Exendin-4 (Ex-4) is a Glucagon-like peptide 1 (GLP-1) receptor agonist approved for the treatment of Type 2 Diabetes (T2DM), which requires daily subcutaneous administration. In T2DM patients, GLP-1 administration is reported to reduce glycaemia and HbA1c in association with a modest, but significant weight loss. The aim of present study was to characterize the site-specific profile and metabolic effects of Ex-4 levels expressed from salivary glands (SG) in vivo, following adeno-associated virus-mediated (AAV) gene therapy in two different animal models of obesity prone to impaired glucose tolerance and T2DM, specifically, Zucker fa/fa rats and high fed diet (HFD) mice. Following percutaneous injection of AAV5 into the salivary glands, biologically active Ex-4 was detected in the blood of both animal models and expression persisted in salivary gland ductal cell until the end of the study. In treated mice, Ex-4 levels averaged 138.9±42.3 pmol/L on week 6 and in treated rats, mean circulating Ex-4 levels were 238.2±72 pmol/L on week 4 and continued to increase through week 8. Expression of Ex-4 resulted in a significant decreased weight gain in both mice and rats, significant improvement in glycemic control and/or insulin sensitivity as well as visceral adipose tissue adipokine profile. In conclusion, these results suggest that sustained site-specific expression of Ex-4 following AAV5-mediated gene therapy is feasible and may be useful in the treatment of obesity as well as trigger improved metabolic profile.

## Introduction

Glucagon-like peptide 1 (GLP-1), a gastrointestinal hormone mainly produced in a nutrient-dependent manner [1], enhances glucose-dependent insulin secretion and inhibits food intake, gastric emptying, and glucagon release, thus promoting the maintenance of normal glucose homeostasis [2]. A small, but significant, defect in oral glucose load and mixed meal stimulated GLP-1 secretion has been observed in T2DM [3], [4]. In T2DM patients, GLP-1 chronic administration reduces fasting and postprandial blood glucose and decreases HbA1c in association with a modest, but significant weight loss [5]. The short half-life of GLP-1, due to rapid inactivation mainly catalyzed by dipeptidyl-peptidase-4 (DDP-4), has engendered interest in the development of more stable longer-acting GLP-1 receptor agonists (GLP-1 RA) for the treatment of T2DM. Exendin-4 (Ex-4), is a natural GLP-1 RA, which because of its molecular structure, is considerably more resistant than native GLP-1 to degradation by DPP-4 [6]. Exenatide (the synthetic form of Ex-4) significantly improves glycaemic control and causes progressive weight loss in T2DM patients, requiring a twice daily subcutaneous administration [7], [8].

Gene therapy offers the possibility of a long-term expression in the treatment of many chronic diseases, including T2DM [9]. Adenoviral and plasmid-based vectors have been used to express GLP-1 RA in several tissues but have not resulted in long term effects either as a result of low or transient expression [10]–[16]. While effective in animal models, the inherent risk profile related to systemic delivery of vectors supported site-specific gene therapeutic approaches as an appealing alternative [17].

Recently, Adeno-associated Viruses (AAVs) have advanced to the forefront of gene therapy, due to their ability to achieve long-term transgene expression in vivo and low immunogenicity [18]–[21]. Several Phase I/II clinical trials support a good overall safety profile for AAV vectors and little associated toxicity in humans [22]–[26]. Over 100 AAV isolates have been reported, biochemical and molecular characterization suggests that some exhibit different tissue tropism, persistence, and transduction efficiency [27]. Among AAVs, serotype 5 (AAV5) has demonstrated enhanced gene transfer activity in lung, eye and CNS as well as rodent salivary glands (SG) [28].

SG are recognized as a useful depot organ in gene therapy, having several important features of other endocrine glands, such as high protein production and ability to secrete proteins into the bloodstream [29]. It has been previously reported that SGs are able to produce pharmacological levels of growth hormone and parathyroid hormone following transduction with recombinant viral vectors [30], [31]. A phase I clinical trial targeting gene transfer to the salivary glands for treating radiation induced xerostomia was initiated and a total of 11 patients have been treated with all reporting tolerance of the vector and procedure [32].

In the present study we have characterized metabolic effects and site-specific secretion profile of sustained Ex-4 expression from the SG of both a polygenic and monogenic animal models of obesity prone to impaired glucose tolerance and T2DM. Ex-4 expression resulted in a significant decrease in weight gain in both models, improved glycemic control and/or insulin sensitivity and visceral adipose tissue adipokine profile, thus suggesting long-term benefit following sustained expression.

**Figure 1:**
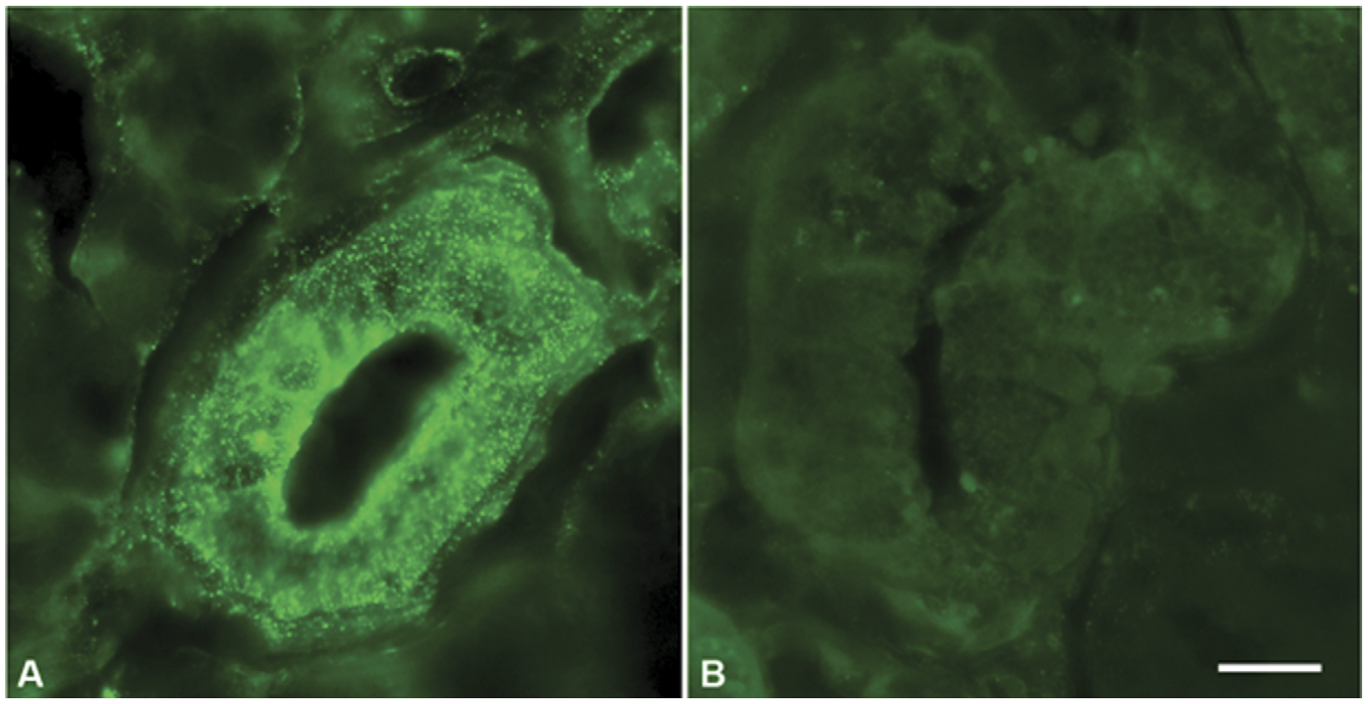
Epifluorescence microscopic image of salivary glands by immunohistochemical assay against Exendin-4, in AAV-5 Exendin-4 treated (Fig. A) and control High Fat-Diet mice (Fig. B); barr  = 20 µm. The immunoreaction products are observed under a Zeiss Axioskop microscope at x40 magnification.

## Materials and Methods

### Construction, Preparation, and Quantification of AAV5 Vectors Carrying the Ex-4 Minigene

Recombinant AAV particles were produced using a four-plasmid procedure as previously described in Di Pasquale G *et al* [33]. The AAV5 vector cassette was designed to contain the cytomegalovirus (CMV) promoter, the mouse nerve growth factor (NGF) signal peptide, which has been shown to mediate secretory expression of polypeptides in vitro and in vivo [34] and the minigene coding for Ex-4. Briefly, semiconfluent human embryonic kidney 293T cells obtained from the American Type Culture Collection (ATCC, Manassas, VA) were transfected by calcium phosphate with four plasmids: an adenovirus helper plasmid (pAd12) containing VA RNA and coding for the E2 and E4 proteins; two AAV helper plasmids containing either the AAV2rep or the AAV5 capsid gene and a vector plasmid including the AAV inverted terminal repeats flanking the Ex-4 expression cassette. The cells were harvested 48 hours post-transfection and a crude viral lysate was obtained after three freeze-thaw cycles. The clarified lysate (obtained by further low-speed centrifugation) was treated with 0.5% deoxycolic acid (DOC) and 100 U/ml DNase (Benzonase) for 30 minutes at 37°C. After purification of viruses by CsCl gradient ultracentrifugation, vector titer was determined by quantitative real-time PCR (qPCR) (Applied Biosystems, Foster City, CA). Immediately before experiments, vector doses were dialyzed against 0.9% NaCl.

### Cell Cultures and *in vitro* Transfection

The generated recombinant AAV5 virus encoding the Ex-4 minigene was then tested in vitro. 293T human renal epithelial cells were grown at 37°C under a 5% CO_2_ humidified atmosphere in Dulbecco’s modified Eagle’s medium supplemented with 10% fetal bovine serum (FBS), 2 mM L-glutamine, 100 U of penicillin/ml, and 0.1 mg of streptomycin/ml. 293T cells were transfected with the AAV5 Ex-4 vector at 10^3^ DNase Resistant Particles (DRP)/ml per cell. Ex-4 levels into the culture medium was assayed using a specific enzyme immunoassay kit (Phoenix Europe GmbH, Germany).

For *in vitro* bioactivity assay, supernatant medium from 293T cells transduced with AAV5 Ex-4 (after 96 hours of incubation) was tested on Chinese hamster ovary cell line obtained from American Type Culture Collection (ATCC, Manassas, VA) stably transfected with rat GLP-1 receptor (CHO-GLP1R) accordingly to previously reported study from Egan JM *et al* [35].

**Figure 2:**
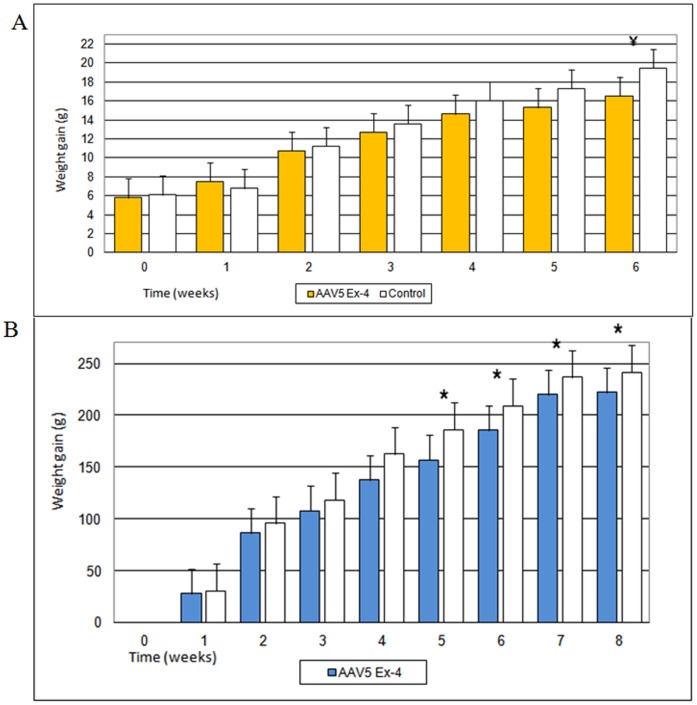
Weight gain of High Fat Diet mice (n = 20) and Zucker fa/fa rats (n = 10) following vector administration. The graphs represent the average values (g) ± Standard Error (SE). Weight gain is expressed as difference (g) between weight at study point and baseline value. (A) Weight gain of High Fat-Diet mice. Each group was composed of ten mice. ¥  =  p<0.01. (B) Weight gain in Zucker fa/fa rats. Each group was composed of five rats. *  =  p<0.05.

**Table 1:** Baseline characteristics of High Fat-Diet (HFD) fed mice and Zucker fa/fa rats (control and AAV5 Ex-4 treated animals).

	Control HFDmice	AAV5 Ex-4 HFDmice	p*	Control Zuckerfa/fa rats	AAV5 Ex-4 Zuckerfa/fa rats	p*
**n**	10	10		5	5	
**Weight (g)**	23.3±1.9	23.0±1.6	p>0.05	290.6±26.2	294.2±28.5	p>0.05
**Fasting glycemia (mmol/L)**	4.6±0.8	4.7±0.6	p>0.05	5.1±0.8	5.3±0.8	p>0.05
**Insulinemia (pmol/L)**	82.1±5.3	86.0±7.2	p>0.05	1456.3±182.2	1528.5±168	p>0.05
**HOMA-IR index (units)**	2.8±0.3	3.0±0.4	p>0.05	55.1±5.6	60.0±7.2	p>0.05
**HbA1c (%)**	<4	<4	p>0.05	4.2±0.1	4.1±0.2	p>0.05
**Glycosuria (number positive)**	0	0		0	0	
**Daily food intake (g)**	2.9±0.8	3.1±0.5	p>0.05	27.8±2.8	29.0±3.0	p>0.05

* Control in comparison to AAV5 Ex-4 HFD mice and control versus AAV5 Ex-4 Zucker fa/fa rats, respectively.

### Animal Models

Animal studies were carried out in strict accordance with the European Communities Council Directive of 24 November 1986 (86/609/EEC) for experimental animal care. The study protocol was approved by the Italian National Health Institute Committee on Animal experiments. All surgeries were performed under anesthesia, and all efforts were made to minimize suffering.

Male 4 week old CD1 mice (n = 20) were purchased from Harlan Laboratories (Udine, Italy), housed at five animals per group and fed High Fat Diet *ad libitum* (Dottori Piccioni Laboratories Srl, Milan, Italy). The HFD supplied 60% of energy as fat and 20% as carbohydrate. Fatty acid composition was as follows: 42.0% saturated fatty acids (palmitic and stearic acids), 43% monounsaturated fatty acids (oleic acid), and 15% polyunsaturated fatty acid (linoleic acid and linolenic acid). The HFD contained 300 mg cholesterol/kg and its energy density was 21.10 kJ/g. The HFD fed mice are recognized as an efficient and robust animal model for obesity, early prone to impaired glucose tolerance and T2DM development [36].

Male Zucker fa/fa rats (n = 10) purchased from Charles River laboratories (Lecco, Italy) were housed in single cage and received standard chow *ad libitum* (Purina Rodents Laboratory Diet). Zucker fa/fa rats are conversely a spontaneous monogenic obesity model, characterized by a missense mutation on the leptin receptor gene [37].

Submandibulary salivary glands of 8 week old HFD mice (n = 10) and of 9 week old Zucker fa/fa rats (n = 5) were transduced by single percutaneous injection of 50 µl of AAV5 Ex-4 (5×10^12^ DRP/ml) vector. Control animals (n = 10 HFD mice; n = 5 Zucker fa/fa rats) received 50 µl of AAV-5 vector devoid of Ex-4 transgene (empty vector). On a weekly basis weight, food, water intake, urine volume, glycaemia were monitored throughout the study. In order to evaluate effects of treatment on short term food consumption, a 120 minutes food intake evaluation, after overnight fasting, was also performed in rats. A fixed amount of standard chow was given in individual cages and rodents food intake (evaluated as the difference between the baseline amount and the residual food, including spillage) was measured every 15 minutes.

An intraperitoneal insulin tolerance test (ITT) was performed in HFD mice, 6 weeks following vector administration. Each animal was fasted for 4 hours. Following intraperitoneal insulin (Humulin R Regular, Lilly) injection (1 UI/kg), blood samples from the lateral tail vein were collected to measure glycaemia at 0, 15, 30, 60, 90 and 120 minutes. The glucose areas under the curve (AUC, mM per minute) were calculated according to formula: AUC_0–120 min_  =  (G_0_+ G_15_ ) x 15/2+(G_15_+ G_30_ ) x 15/2+(G_30_+ G_60_ ) x 30/2+(G_60_+ G_120_ ) x 60/2.

Blood samples were withdrawn at baseline and week 6 in HFD mice in order to detect Ex-4, glycaemia, insulinemia, HbA1c, leptin and adiponectin circulating levels and at 0, 4, and 8 week in Zucker fa/fa rats to detect Ex-4 and glucose values. HbA1c and insulinemia were determined at baseline and 8 weeks after vector administration. Blood samples were obtained through jugular sampling conducted in isoflurane-anesthetized animals. At study end, rats and mice were respectively euthanized by CO_2_ (80%) inhalation.

SG, liver, spleen, and pancreas tissues were collected for DNA extraction and vector distribution assay.

**Figure 3:**
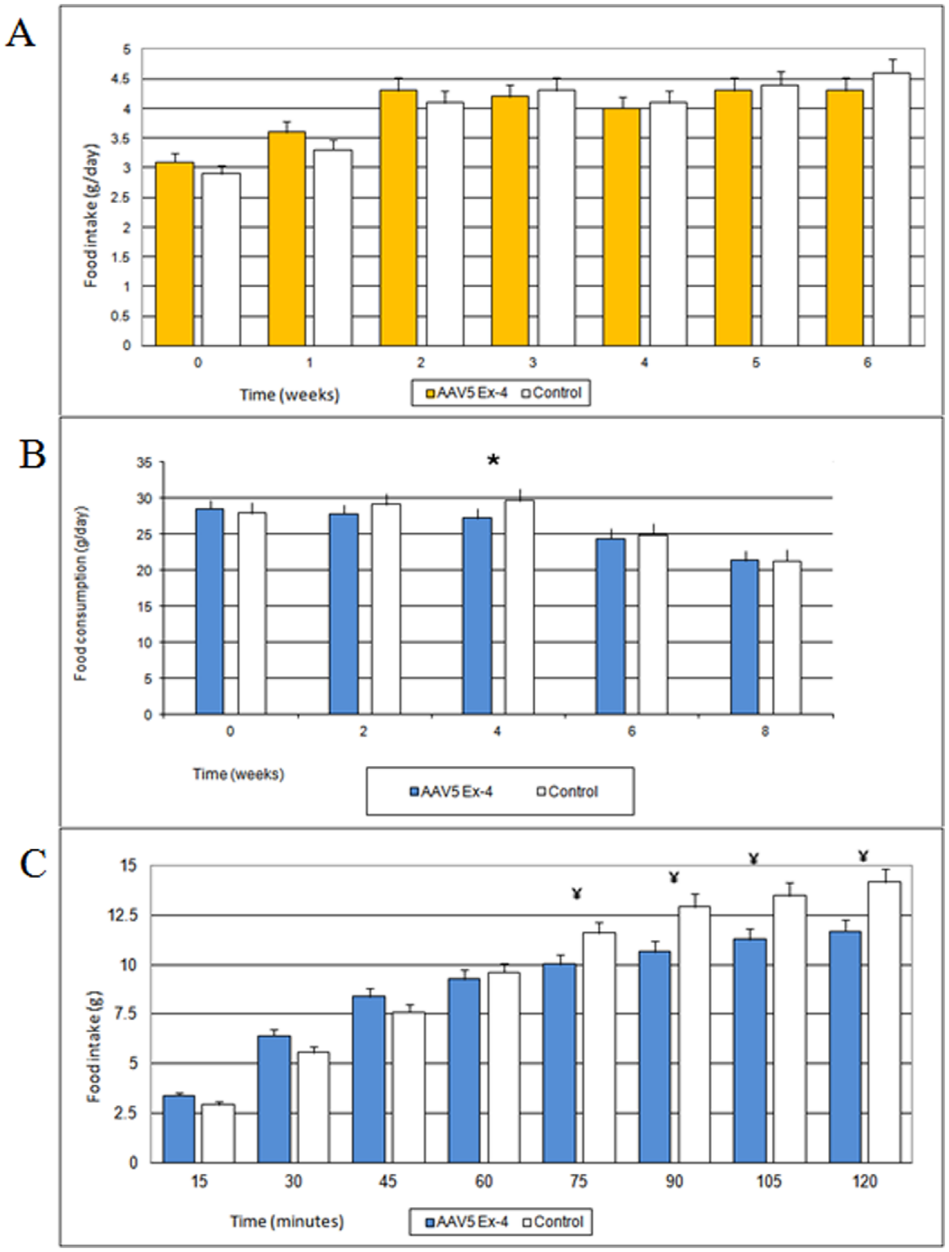
Daily and short term food intake (average values ± Standard Error). (A) Daily food intake in High Fat-Diet mice. Each group was composed of ten mice. (B) Daily food intake in Zucker fa/fa rats. Each group was composed of five rats. * p<0.05. (C) Short term food intake in Zucker fa/fa rats 4 weeks after vector administration. Each group was composed of five rats. ¥  =  p<0.01.

### Ex-4 Assay

Ex-4 exhibits 53% structural homology to native GLP-1. Circulating Ex-4 levels were determined by a specific enzyme immunoassay kit (Phoenix Europe GmbH, Germany), unable to detect endogenous GLP-1 values, according to the manufacturers’ instructions. Minimum detectable concentration was 2.6 pmol/L.

### Vector Biodistribution

In order to assess vector biodistribution at the end point of the study, a DNA isolation kit was used to purify total genomic DNA from SG, liver, spleen and pancreas (Wizard DNA purification kit, Promega Corporation, Madison, WI, USA). Quantitative PCR amplification (20 µl final volume) of genomic DNA (100 ng) was performed with ABI PRISM 7700 Sequence Detection System (Applied Biosystems, Foster City, CA) by using the SYBR Green PCR Master Mix and a specific 5′ and 3′ primer pair appropriate (0.3 µM; CMV forward 5′-CATCTACGTATTAGTCATCGCTATTACCAT- 3′, CMV reverse 5′-TGGAAATCCCCGTGAGTCA-3′) for CMV promoter. Amplification and detection were performed with ABI Prism 7700 Sequence Detection System (Applied Biosystems, Foster City, CA). A PCR cycling reaction involved an initial hold at 95° for 10 minutes, followed by cycling conditions of 95°C for 15 seconds, 60°C for 1 min for 40 cycles. The viral DNA in each sample was quantified by comparing the fluorescence amplification profiles with a set of DNA standards using AAV5 vector and 100 ng of genomic DNA of untreated animals for each specific tissue. Each measurement was carried out in duplicate. Data are expressed in copies of AAV5 for 100 ng of genomic DNA.

**Figure 4:**
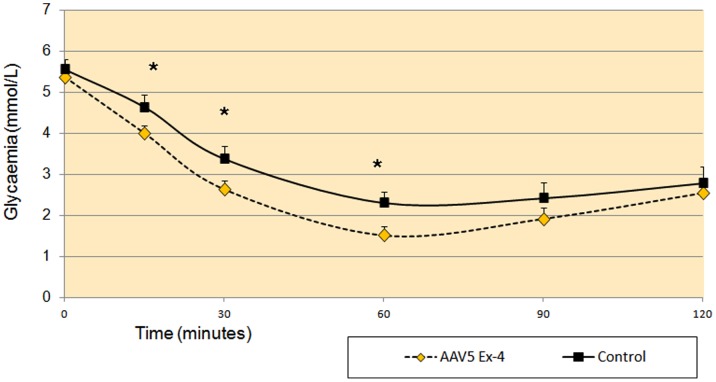
Intraperitoneal insulin tolerance test at week 6, in High Fat-Diet mice. Each group was composed of ten mice and the graphs represent the average glycaemic values (mmol/L) ± Standard Error (SE). *  =  p<0.05.

### Salivary Glands Immunohistochemical Assay

At the end of the study, SG were removed from treated (n = 5) and control (n = 5) HFD mice, fixed in 4% paraformaldehyde for 24 hours at room temperature, cryoprotected in 30% sucrose in phosphate-buffered saline (PBS) for approximately 12 hours at 4°C and then embedded in Killik cryostat embedding medium (Bio-Optica, Milan Italy). Cryosections, 10 µm thick, were collected on polylysine-coated slides.

The slides were pre-incubated in 0.5% Triton (Sigma Aldrich, Milan, Italy) and 1.5% Bovine Serum Albumin (BSA) (Sigma Aldrich, Milan, Italy) in PBS for 15 minutes at room temperature to saturate non specific sites. Then the sections were incubated 24 hours at 4°C with primary antibody against Ex-4 (Phoenix Europe GmbH, Germany) at final dilution of 1∶50.

Subsequently, the sections were incubated with an Alexa Fluor 488 secondary Donkey anti Rabbit antibody (Invitrogen, San Diego, CA, USA) at a final dilution of 1∶333 for 2 hours at room temperature. The immunoreaction products were observed under an epifluorescence Zeiss Axioskop microscope (Zeiss, Germany) at x40 magnification.

### Adipokines Circulating Levels Assay

Serum leptin and adiponectin levels were assayed only in the polygenic model of obesity prone to im paired glucose tolerance and T2DM such as HFD mice using commercially available kit according to manufacturer’s instructions. Sandwich enzyme immunoassay (ELISA) was used for the quantitative measurement of mouse proteins (Biovendor, Heidelberg, Germany and B-Bridge International Inc., CA, USA, for leptin and adiponectin respectively). Intra- and inter-assay coefficient of variation were less than 5%.

### Visceral Adipose Tissue Adipokines Profile: RNA Extraction and Real Time PCR Determinations

Total RNA was extracted from 50 mg of mice visceral adipose tissue. Briefly, tissue samples were collected, immediately snap frozen in liquid nitrogen and disrupted by homogenization in QIAzol Lysis Reagent using the TissueLyser (QIAGEN GmbH, Hilden, Germany). RNA was extracted using RNeasy Lipid Tissue Mini Kit (QIAGEN GmbH, Hilden, Germany) according to the manufacturer’s instructions. One µg of RNA was treated with TURBO DNA-free™ DNase Kit (Ambion, Inc, Austin, TX, USA) and reverse-transcribed into cDNA for 1 h at 37°C in a 50 µl reaction containing 1× RT buffer, 150 ng random hexamers, 0.5 mmol/l dNTPs, 20 units of RNAsin Ribonuclease Inhibitor (Promega Corporation, Madison, WI, USA) and 200 units of M-MLV RT (Promega Corporation, Madison, WI, USA).

Real Time quantitative PCR was carried out on DNA Engine Opticon™ 2 Continuous Fluorescence Detection System (MJ Research, MA, USA), using Platinum® SYBR® Green qPCR SuperMix-UDG (Invitrogen Corporation, CA, USA) and 300 nM specific primers for each gene : 18 s forward 5′-CGG CTA CCA CAT CCA AGG AA-3′, reverse 5′-GCT GGA ATT ACC GCG GCT-3′; leptin forward: 5′-TCC AGA AAG TCC AGG ATG ACA C-3′, reverse: 5′-CAC ATT TTG GGA AGG CAG G-3′; adiponectin forward: 5′-ACA ATG GCA CAC CAG GCC GTG A-3′, reverse: AGC GGC TTC TCC AGG CTC TCC TTT-3′. Each cDNA sample was assayed in duplicate and a no template control was included in every reaction. For each sample, gene expression values were normalized by 18s RNA content and reported as AU ratio.

### Blood and Urine Analysis, HOMA-IR Index

Glycaemic values were determined in the morning, after overnight fasting. Blood was obtained via tail vein and tested, using an Accu-Chek Aviva Nano meter (Roche). HbA1c percentage values were measured on 5 µl of whole blood using an A1CNow ^+^ test Kit (Bayer). Insulin levels were determined using a commercially available insulin RIA kit (Linco Research, Inc., St. Charles, MO); the intra-assay coefficient of variation was 1.4%. Insulin resistance was evaluated by the homeostasis model assessment of insulin resistance (HOMA-IR) index calculated as: (fasting glycaemia, mmol/L x fasting serum insulin, pmol/L/135) [38]. Urine analysis was performed by a colorimetric method (AUTION Sticks 10TA; Arkray, Inc, Kyoto, Japan) in order to detect glucose levels.

### Statistical Analysis

The statistical significance of differences between experimental and control groups was analyzed by Student’s t-test. p<0.05 was considered statistically significant. Values are presented as mean ± standard error (SE).

**Table 2:** Final characteristics of High Fat-Diet (HFD) fed mice and Zucker fa/fa rats (control and AAV5 Ex-4 treated animals).

	Control HFDmice	AAV5 Ex-4HFD mice	p*	Control Zuckerfa/fa rats	AAV5 Ex-4 Zuckerfa/fa rats	p*
**n**	10	10		5	5	
**Weight gain (g)**	19.5±1.9	16.5±2.7	**p<0.01**	241.4±22.5	222±23.4	**p<0.05**
**Fasting glycemia (mmol/L)**	4.9±0.9	4.8±0.7	p>0.05	5.7±0.4	5.6±0.5	p>0.05
**Insulinemia (pmol/L)**	150.6±6.1	126.3±4.0	**p<0.01**	3862.9±320.3	3164.8±265.0	**p<0.05**
**HOMA-IR index (units)**	5.5±0.4	4.5±0.4	p>0.05	163.1±15.0	131.2±11.2	**p<0.05**
**HbA1c (%)**	4.2±0.2	4.1±0.2	p>0.05	5.0±0.1	4.7±0.1	**p<0.05**
**Glycosuria (number** **positive)**	0	0		4	0	**p<0.05**
**Daily food intake (g)**	4.3±0.3	4.6±0.3	p>0.05	21.2±2.1	21.3±1.9	p>0.05

* Control in comparison to AAV5 Ex-4 HFD mice and control versus AAV5 Ex-4 Zucker fa/fa rats, respectively.

## Results

### Expression of Immunoreactive and Biologically Active Ex-4 *in vitro* and *in vivo*

In order to facilitate processing and secretion, Ex-4 coding sequence was linked to the secretory signal peptide from nerve growth factor (NGF), which was modified to be cleaved by the furin protease. Initial in vitro experiments suggested the chimeric protein could be secreted from cells in culture. Media collected from AAV5 Ex-4 transduced 293T cells produced an average of 38.3±10.4 pmol/L when assayed for Ex-4 biological activity on Chinese hamster ovary cell line stably transfected with rat GLP-1 receptor (CHO/GLP-1R). The furin overexpression by transfection of a plasmid encoding the protease, increased the active Ex-4 in the medium to 75.6±11.0 pmol/L.

Ex-4 secretion was evaluated *in vivo* in two different animal models of obesity prone to impaired glucose tolerance and T2DM, following single 50 ul (5×10^12^ DRP/ml) vector administration into their SG. Our initial *in vivo* experiments suggested that like erythropoietin (27), NGF-Ex-4 can be produced by the salivary glands and will traffic through the cell via the endocrine pathway resulting in circulating serum levels. In AAV5 Ex-4 HFD mice (n = 10), Ex-4 levels averaged 138.9±42.3 pmol/L at week 6, when assayed by a specific Enzyme Immunoassay (EIA) kit. In AAV5 Ex-4 Zucker fa/fa rats (n = 5), mean circulating Ex-4 level was 238.2±72 pmol/L at week 4 and increased to 3.25 nmol/L at week 8. In control animals, average circulating Ex-4 levels resulted less than 2.6 pmol/L, thus above detection limit, at week 6 in mice and both at week 4 and 8 in rats.

Biological activity of Ex-4 was also confirmed on CHO/GLP-1R cells (data not shown).

### Vector Biodistribution and Expression *in vivo*

In order to assess vector biodistribution, DNA samples were collected from the SG, liver, spleen and pancreas of HFD mice (n = 5 AAV5 Ex-4 treated and n = 5 naïve mice) at the end of the study and vector copy number was determined by quantitative Polymerase Chain Reaction (qPCR) amplification using specific primers for the CMV promoter. Naïve animals yielded background levels of 55±29 copies/100 ng of DNA extracted from SG. In AAV5 Ex-4 HFD mice, a 60-fold increase in vector copy number was detected in SG (3551±1618 copies/100 ng of DNA). The vector copy number detected in other tissues such as liver (154±56 copies/100 ng of DNA), spleen (65±23 copies/100 ng of extracted DNA) and pancreas (104±47 copies/100 ng of DNA) were at or near background levels.

Expression was also confirmed by immunohistochemical staining for Ex-4 in SG sections from AAV5 Ex-4 (n = 5) and control (n = 5) HFD mice euthanized at week 6. Ex-4 expression is observed only in the AAV5 Ex-4 treated group. Only salivary ductal cells revealed positive staining, which is consistent with the tissue tropism of the AAV5 vector (**Figure 1B**).

### Reduced Weight Gain in Ex-4 Treated HFD Mice and Zucker fa/fa Rats

At baseline HFD mice treated with AAV5-Ex 4 vector were not significantly different from control animals with respect to weight, fasting glucose and insulinemia, HOMA-IR index, HbA1c, glycosuria or daily food intake (**Table 1**). Both case and control animals received High Fat diet *ad libitum* and continued to gain weight throughout the study. However, at termination of the study (week 6), AAV5 Ex-4 HFD mice had a significantly lower weight gain in comparison to control animals (**Figure 2A**).

Zucker fa/fa rats are a spontaneous monogenic model of obesity as a result of a dysfunctional leptin receptor. At baseline, AAV5 Ex-4 rats also were not significantly different from control animals with respect to weight, fasting glucose and insulinemia, HOMA-IR index, HbA1c levels, glycosuria, and daily food intake (**Table 1**). These animals received standard chow *ad libitum* throughout the study, which was terminated 8 weeks after vector administration. At week 5, AAV5 Ex-4 rats had a statistically significant lower weight gain compared with control animals, which persisted for the duration of the study (**Figure 2B**).

Differences in daily food intake between treated and control animals were detected only transiently in rats (**Figure 3B**), but not in mice (**Figure 3A**). In AAV5 Ex-4 rats, daily food consumption was significantly lower at week 4 compared with controls. Similarly, short-term food intake monitoring was also reduced in the AAV5 Ex-4 treated rats compared with control animals by 75 minutes (**Figure 3C**).

### Reduced Leptin Levels in AAV-5 Ex-4 Treated Mice

In addition to reduced weight gain, AAV5 Ex-4 treated mice had significant lower circulating levels of leptin (week 6) compared with control animals (2.24±0.39 versus 5.89±1.07 ng/ml; p<0.01). In contrast, no significant difference in adiponectin levels were observed (9.75±0.69 versus 10.57±0.97 mg/l; P = NS). The reduction in leptin circulating levels correlated with a significant reduction in visceral adipose tissue leptin mRNA expression compared with control animals (3.43±0.48 versus 8.28±0.72 Arbitrary Unit, AU; p<0.01). No difference in visceral adipose tissue adiponectin mRNA expression (8.28±0.72 versus 8.95±1.8 AU; P = NS) was detected.

### Improved Glycemic Control and/or Insulin Sensitivity both in Treated HFD Mice and Zucker fa/fa Rats

Mice feed a high fat diet will develop T2DM after 12 weeks. Accordingly, the model animals exhibited throughout the study a mild increase of glycemia and HbA1c, high serum insulin levels and HOMA-IR index. In order to better understand early effects of AAV5 Ex-4 treatment on the development of insulin resistance, insulin tolerance was tested (ITT) following an intraperitoneal insulin injection. At week 6, AAV5 Ex-4 mice showed a greater insulin-induced reduction in glycaemia 15, 30 and 60 minutes following injection compared with control mice (**Figure 4**). The corresponding glucose AUC over 120 minutes was significantly in AAV5 Ex-4 mice in comparison to control animals (333.7±14.2 vs. 378±12.3 mM per minute; p<0.05).

No significant difference was observed in fasting glycaemia, glycosuria or HbA1c values throughout the study. At the end of the study, AAV5 Ex-4 mice showed significant lower fasting insulin circulating levels and HOMA-IR index compared with control animals (**Table 2**).

On the other hand, significant reductions in HbA1c levels and glycosuria were observed in Zucker fa/fa rats. AAV5 Ex-4 treatment resulted in significantly lower HbA1c level as compared with control animals (4.7±0.1 versus 5.0±0.1%; p<0.05). Glycosuria was detected in 4 control rats 8 weeks after vector administration. No glycosuria was reported in treated rats. Accordingly to the low hypoglycaemic risk profile of Ex-4, no significant difference in fasting glycaemia was observed between treated and control rats during the study. Moreover, a statistically significant difference in fasting insulinemia and HOMA-IR index was detected between treated and control animals at the end of the study (**Table 2**).

## Discussion

GLP-1 RA are a very interesting therapeutic approach for the treatment of T2DM, showing a remarkable efficacy on glycaemic control and beneficial effects on body weight [39], [40]. A phase II clinical trial has shown the potential efficacy and safety of GLP-1 RA in the treatment of obesity [41], although this disease is not among the approved indication. Wider use of GLP-1 RA is presently limited by their cost, and need for multiple subcutaneous administration. Adenoviral and plasmid DNA mediated gene therapy can direct the expression of GLP-1 RA in tissue not physiologically intended for secretion [10]–[16] achieving long term metabolic effects through high vector doses systemic administration either via intravenous or intraperitoneal injection. Both systems demonstrated short-term efficacy on metabolic improvement and required high vector doses and/or systemic administration. Recently, Voutetakis *et al* reported that an adenoviral-mediated transduction of SG with a vector encoding GLP-1, can induce short-term moderate reduction of blood glucose in a murine model of diabetes [29]. Not surprisingly, although those approaches were shown to reduce blood glucose, no effect on HbA1c levels has ever been reported, confirming that the therapeutic efficacy was not sustained.

Our study shows for the first time a sustained secretion of Ex-4 at pharmacological levels. These circulating levels are several fold higher than reported for endogenous human GLP-1 after a meal (40 pmol/l) [42] and Ex-4 steady-state values attained in human clinical studies with 10 µg injected exenatide (50 pmol/l) [43]. This sustained Ex-4 secretion resulted in a significant reduction in weight gain, which persisted throughout the study. The mechanism underlying the effect of Ex-4 on body weight is still controversial and likely related to a peripheral action on gastric motility and/or a direct effect on the hypothalamic region involved in the regulation of eating behaviour. Effects on daily food intake between treated and control animals were detected only transiently in rats, which showed a reduced meal size, suggesting enhancement of satiety. It should be noted that limitations on the accuracy of measuring food intake could have prevented detection of a difference in food intake associated with changes in body weight over the long-term.

A reduction of fasting insulin levels, together with an improvement of HOMA insulin resistance index (based on fasting insulin and glucose) was observed in treated rats and mice, suggesting that Ex-4 secretion improves insulin sensitivity; this was confirmed by the results of insulin tolerance test in HFD mice. Although weight loss could explain the enhancement of insulin sensitivity, a direct insulin-sensitizing effect of Ex-4 is also possible. Alternatively, the improvement in insulin sensitivity could be due to the inhibition of glucagon secretion.

Although the effect of Ex-4 expression on body weight could have contributed to the improvement in glycaemic control, it is very likely that direct action of Ex-4 on insulin and glucagon secretion, and possibly insulin resistance, played a major role.

Furthermore, some limitations of present study should be recognized. 14 week HFD mice and 17 week Zucker fa/fa rats are more experimental model of obesity, and insulin resistance than models of overt diabetes, as indicated by fasting glycemic values throughout the study. Therefore, we performed the insulin sensitivity test in the HFD mouse, because of its greater similarity to polygenic pathogenesis of human obesity compared with Zucker fa/fa rat. However, glycemic control was evaluated through HbA1c (as a surrogate marker), for fasting glycemia and glycosuria detection in both animal models.

This study suggests an alternative approach to delivering Ex-4 is possible and can reduce weight gain as well as trigger improved metabolic function in two animal models. Although Ex-4 expression has shown metabolic benefits there is concerns about the long-term safety of this drug and its effect on inducing tumors in rodents [44], which has not been reported in humans. This affect maybe the result of the high bolus delivery of Ex-4 necessary with injection delivery and is not observed with gene therapy based delivery, which can maintain a constant level of expression. Other studies have demonstrated that transgene expression in rodents can last of the life of the animal following gene transfer to several tissues including salivary glands [29]. Although, no adverse effects of sustained expression were noted in either animal model over the 2 month period of this study additional long-term studies would support the long term safety of this drug.
